# Her2-Positive and Microsatellite Instability Status in Gastric Cancer—Clinicopathological Implications

**DOI:** 10.3390/diagnostics11060944

**Published:** 2021-05-25

**Authors:** Ana Bermúdez, Isabel Arranz-Salas, Silvia Mercado, Juan A. López-Villodres, Virginia González, Francisca Ríus, María V. Ortega, Carmen Alba, Isabel Hierro, Diego Bermúdez

**Affiliations:** 1 Department of Anesthesiology, Nuestra Señora de Valme University Hospital, 41014 Seville, Spain; abermudez@uma.es; 2 Department of Human Physiology, Human Histology, Anatomical Pathology and Physical Education, University of Malaga, 29010 Malaga, Spain; iarranz@uma.es (I.A.-S.); smercad@uma.es (S.M.); jantoniolv@uma.es (J.A.L.-V.); mariavi@uma.es (M.V.O.); mcalba@uma.es (C.A.); 3 Unit of Anatomical Pathology, Virgen de la Victoria University Hospital, 29010 Malaga, Spain; ihierro@uma.es; 4 Unit of Anatomical Pathology; Montilla Hospital, 14550 Montilla, Spain; virginia.gonzalez.zafra.sspa@juntadeandalucia.es; 5 Department of Public Health and Psychiatry, University of Malaga, 29010 Malaga, Spain; rius@uma.es

**Keywords:** gastric cancer, molecular classification, HER2, microsatellite instability, clinicopathological features

## Abstract

Gastric cancer (GC) is one of the leading causes of cancer-related death. The combination of new molecular classifications with clinicopathological data could contribute to the individualization of patients and to the development of new therapeutic strategies. We examined the various associations in two molecular types of GC: HER2-positive (human epidermal growth factor receptor 2) and microsatellite instability (MSI), assessing their influence on treatment and prognosis. A retrospective study of 142 GC patients was performed with molecular characterization through HER2 overexpression and DNA repair protein expression for MSI. The percentage of HER2-positive tumors was 13.4%, predominantly in men. Correlations were found with intestinal type, metastases, advanced stages and chemotherapy. Almost 75% of HER2-positive patients died. MSI occurred in 16.2%, associated with advanced age, female sex, distal location and intestinal type. These patients had few metastases and low stages. The percentage of deaths was higher among MSI patients who received perioperative chemotherapy. The determination of HER2 and MSI status in GC is important for their association with specific clinicopathological features and for their prognostic and predictive value.

## 1. Introduction

Gastric cancer (GC) is the fifth most common malignancy worldwide and a leading cause of cancer-related death [1,2,3,4,5,6,7,8,9,10,11,12,13]. Most patients are diagnosed at advanced stages of the disease, making surgery difficult and the prognosis poor [7,8,14,15,16]. Unlike other tumors, there have been no major advances in GC in terms of survival, which is still less than 30% at 5 years [1,5,6,7,8,17,18]. Special mention should be made of HER2-positive cases in which specific treatment with HER2 inhibitors resulted in the first improvements in survival of patients with GC [1,6,9,19,20,21].

Although different anatomical and histological classifications of GC have been proposed, they lack clinical utility, as they have no prognostic or predictive value [1,4,5,6,7,19]. Furthermore, it is important to consider cancer not as a single process, but as a set of molecular alterations that can offer different therapeutic targets and treatment strategies, resulting in important advances in other tumor processes [1,22]. GC is a complex, heterogeneous and multifactorial disease [4,7], and its molecular characterization could establish different types to enable the individualization of patients; hence, the importance of the new molecular classifications of GC [8,12,22,23,24,25,26].

The Singapore-Duke group first distinguished two types of GC [22] and, subsequently, three types [27]. In 2014, The Cancer Genome Atlas (TCGA) group established four molecular categories of GC: chromosomal instability (CIN), microsatellite instability (MSI), genomically stable (GS) and Epstein-Barr virus-positive (EBV) [28]. This classification was a major advance as it reflects tumor biology and can be associated with certain clinicopathological data [1,4,29]. It is considered of great importance for GC diagnosis [1,4] and for the selection of targeted therapeutic agents [7,9,19]. Soon after, the Asian Cancer Research Group (ACRG) [30] identified four types: mesenchymal, MSI, microsatellite stable (MSS) TP53+ and microsatellite stable TP53-, which correlate with the prognosis, as well as with different molecular and disease progression patterns [9,29].

Reviews of GC biomarkers have also been conducted [6,17], although to date only HER2 and PD-L1 (Programmed Death-ligand 1) are able to predict treatment response [9,29,31]. HER2 is the only marker routinely evaluated and widely used for targeted therapy in GC [2,15,16,17,19]. In addition, an immune molecular classification based on tumor microenvironment has recently been proposed [32].

The TCGA classification is the most widely used [1,3,4,5,6,8,9,14,19,23,33,34], though there is still a need for a classification that is able to combine molecular patterns with clinical and pathological data for prognostic and predictive utility [4,5,8,33]. Nevertheless, the success obtained with anti-HER2 therapies [1,6,21], the use of immune checkpoint inhibitors in some types of GC, and new molecular markers open the door to a combination of classical chemotherapy, immunotherapy and molecular therapy, which could improve outcomes and survival [9,12,19,25,31,35]. Pathology laboratories could play an important role in these studies and in the correlation between clinicopathological data with molecular types from a multidisciplinary approach involving oncologists, surgeons and pathologists [21,33].

HER2 is a GC subtype included in the CIN category of the TCGA [28]. It is a proto-oncogene, a member of the HER family [18,24], encoding a transmembrane receptor with tyrosine kinase activity that regulates proliferation, survival, differentiation, migration and other cellular responses to cancer [5,6,17]. Overexpression of HER2 induces malignant transformation and metastasis [24]. HER2-positive GC occurs in a variable percentage (10–30%) and is more commonly associated with proximal location, intestinal type, men and advanced age at diagnosis [1,5,6,7,9,17,21,36,37,38]. Primarily identified in breast cancer, its predictive value has also been established in GC [17]. In addition to trastuzumab, other HER2 inhibitors are being investigated to prevent resistance [1,7,9,17,19,39]. Although most studies indicate a worse prognosis in HER2-positive GC, this aspect remains controversial [1,5,6,17,19,20,24,36,38]. Microsatellites are repetitive sequences of between one and six nucleotides, located in the DNA, in which genomic instability can occur due to a failure in the mismatch repair (MMR) system performed by DNA repair proteins [4,5,6,7,14,17,19,37]. MSI accounts for 10–30% of GC, occurs mainly in the intestinal type, at advanced ages, in women and in the distal stomach. It is associated with low tumor stages, limited lymph node involvement, absence of metastasis and longer survival than in MSS tumors [1,2,4,7,19,23,29,30,33,40]. MSI determination is used for prognostic and therapeutic purposes [6,17,19]. Survival in MSI GC has been reported to be higher in patients treated with surgery alone, with a worse prognosis in those treated with neoadjuvant chemotherapy [1,4,7,40,41,42,43].

It has been suggested that the new molecular classifications of GC in combination with clinicopathological parameters would make it possible to distinguish different groups of patients, improving gastrointestinal oncology and bringing us closer to precision medicine [1,14,26].

Our aim was to correlate HER2-positive and MSI GC subtypes with clinicopathological data to assess whether this could influence treatment or prognosis.

## 2. Materials and Methods

### 2.1. Sample Selection

This was a retrospective study of all patients diagnosed with GC treated with total or partial gastrectomy at Virgen de la Victoria University Hospital in Malaga (Spain), in the period 2008–2015 (*n* = 142). Patients were excluded when: (1) we could not access clinical and/or pathological data; (2) the tumor tissue samples obtained were insufficient for the study (thickness less than 50 µm).

### 2.2. Clinical and Pathological Data

The clinicopathological data collected were as follows: age, sex, location, pathological diagnosis, histological type according to the Lauren classification [44], degree of differentiation according to the World Health Organization [45], TNM classification and stage [46], lymphatic, vascular and perineural involvement, perioperative chemotherapy (administered or not) and survival (patient living or deceased).

Tissue samples were formalin-fixed and paraffin-embedded, sectioned, stained with hematoxylin-eosin and assessed by light microscopy.

### 2.3. Molecular Data

For immunohistochemical analysis, six 4-µm slices were obtained from each block. Molecular analysis of HER2 was performed by protein overexpression/quantification and of MSI by DNA repair protein expression (MMR).

HER2 protein determination was carried out using the Dako Herceptest™ kit (Rabbit Anti-Human HER2 Protein antibody) (Dako Denmark A/S, Glostrup, Denmark) on the automated DAKO Autostainer platform. A breast carcinoma with 3+ protein expression for HER2 was selected as a positive control and a breast carcinoma with 0 protein expression for HER2 was selected as a negative control. Cases with Histoscore 3+, in which intense basement membrane and basolateral staining was observed in more than 10% of the cells, were considered positive overexpression.

Monoclonal antibodies directed against MLH1, MSH2, MSH6 and PMS2 proteins, processed in a DAKO Autostainer, were used for the MSI study. Tonsil tissue was used as a positive control. For each protein, two categories were assessed: preserved expression and loss of expression, depending on whether or not there was any nuclear staining. When this was absent in all tumor cells the tumor was considered to be MSI.

### 2.4. Sample Size and Statistical Analysis

A minimum of 51 patients were required to detect a significant percentage difference of at least 17% between any two groups in the HER2 and MSI study with all other variables, with 95% confidence and a power of 80%.

A descriptive analysis of the data was performed, calculating the usual descriptive data for quantitative variables and frequency tables for qualitative variables. For the inferential analysis concerning the presence or absence of MSI and HER2, Student’s *t*-test was used for quantitative variables and Pearson’s chi-squared test for qualitative variables, after checking homoscedasticity. The mean and standard deviation in the case of Student’s *t*-test and percentages of MSI and HER2 were used as descriptors, according to the modalities of the different study variables. Logistic regression analysis was used to examine the relationship between living versus deceased with MSI and chemotherapy. Calculations were performed with SPSS version 24.0, IBM Corp., Armonk, NY, USA, and the difference was considered statistically significant at *p* < 0.05.

## 3. Results

### 3.1. Study Group. Clinicopathological Features

**Figure 1:**
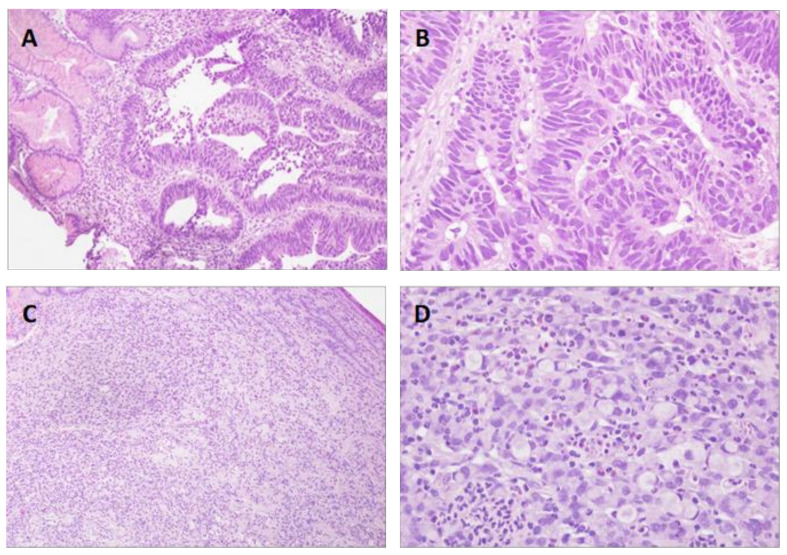
Histological types of gastric cancer according to the Lauren classification. (**A**,**B**): Intestinal type. Normal gastric mucosa and tumor nests with intestinal adenocarcinoma morphology are observed ((**A**), 100×; (**B**), 400×). (**C**,**D**): Diffuse type. There is diffuse infiltration of tumor cells, some with signet ring cell appearance ((**C**), 100×; (**D**), 400×). Hematoxylin-eosin.

The study included 142 patients who underwent gastrectomy for GC, whose clinical, pathological and molecular features, the latter in relation to HER2 and MSI, are shown in Table 1. There were 19 HER2-positive cases (13.4%) and 23 MSI cases (16.2%). The mean age was 65.41 years, with 89 men and 53 women. The tumor was mainly located in the corpus (40.8%) and antrum (41.5%). The histological type was intestinal in 53.5% and diffuse in 40.1% (Figure 1). There were 104 cases of adenocarcinoma and 34 of signet ring cell carcinoma, with a predominance of G3 differentiation (53.6%). TNM classification showed a higher frequency of T3 (40.1%) and T4 (30.3%), with no regional lymph node involvement in 38% of the cases and no distant metastasis in 90.8%. Most tumors were stage II (33.1%) and III (32.4%). There was lymphatic involvement in 45.1%, vascular involvement in 41.5% and perineural involvement in 33.8%. Chemotherapy was administered to 43 patients (30.3%), and at the time of the study 56 patients (39.4%) were living.

### 3.2. HER2-Positive Status and Clinicopathological Features

**Figure 2:**
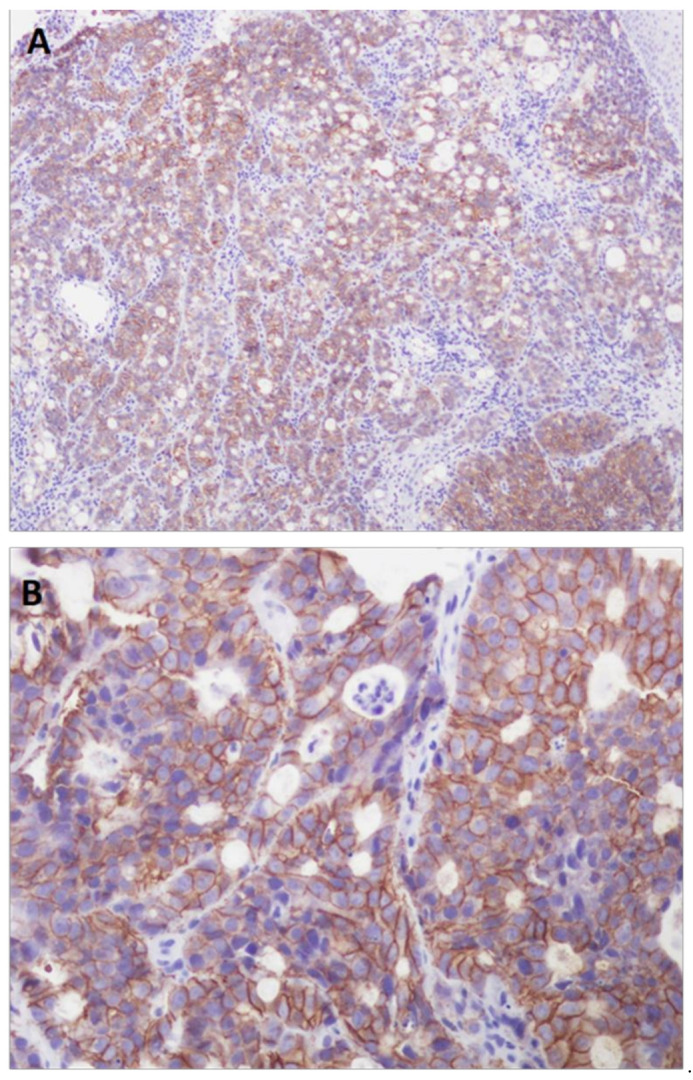
HER2-positive gastric cancer (Histoscore 3+). (**A**,**B**): Immunohistochemical technique showing intense basement membrane and basolateral staining in more than 10% of the cells ((**A**), 100×; (**B**), 400×)).

Table 2 shows the clinical and pathological features of the 142 GC patients and the correlation with HER2 status (Figure 2). The mean age of HER2-positive cases was slightly lower than that of negative cases (61 versus 66 years), with HER2-positive status in 14.6% of men and 11.3% of women. The association with tumor location was statistically significant (*p* = 0.041), with HER2 positivity in 4.3% of GCs located in the cardia, 100% in the fundus, 13.8% in the corpus and 5.3% in the antrum. The correlation with histological type was also significant (*p* = 0.013), with HER2-positive accounting for 21.1% of the intestinal-type tumors and 11.1% of the mixed-type tumors.

Eighteen cases of adenocarcinoma were HER2-positive, as were 17.4% of G2 tumors, 14.9% of G3 tumors, 19.3% of T3 tumors, and 16.27% of T4 tumors, with lymph node involvement in 14 of the 19 cases. There was a significant association with metastases (*p* = 0.005), such that although 14 HER2-positive patients had no metastases, of the 13 patients in the series who had metastases, five were HER2-positive. The majority of cases were classified as stage III and IV (*p* = 0.012). HER2 positivity was found in 17.2% of patients with lymphatic involvement, 16.9% with vascular involvement and 14.6% with perineural involvement. The administration of adjuvant chemotherapy was significantly correlated (*p* = 0.005), with 25.6% of the patients receiving chemotherapy being HER2-positive. Of the total deceased, 16.3% had HER2-positive tumors, which corresponded to 14 of the 19 HER2-positive cases.

### 3.3. MSI Status and Clinicopathological Features

**Figure 3:**
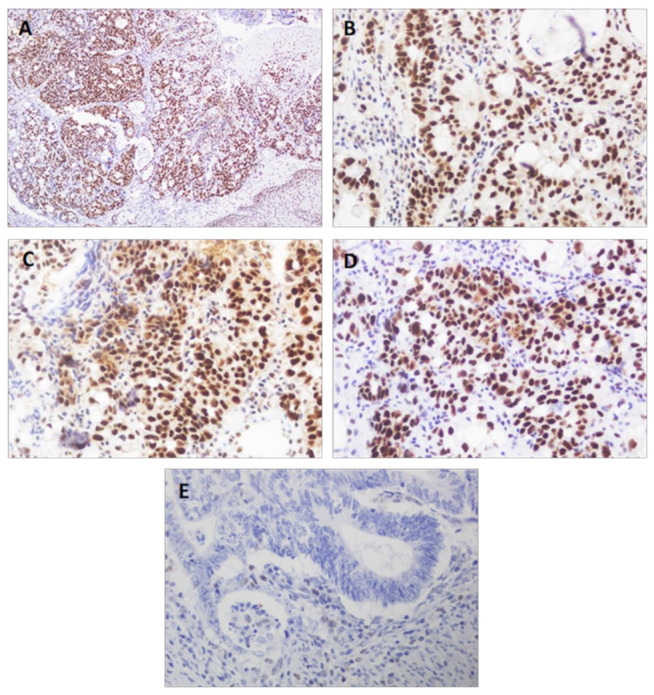
Gastric cancer. Immunohistochemical staining for DNA repair proteins. Preserved immuno-expression of MLH1 ((**A**), 100×), MLH2 ((**B**), 400×), MSH6 ((**C**), 400×) and PMS2 ((**D**), 400×) proteins. (**E**): Loss of MLH1 expression in tumor cells (positive internal control in accompanying lymphocytes) (400×).

Table 3 provides the clinical and pathological features of the 142 GC patients and the correlation with MSI status (Figure 3). Age showed a statistically significant correlation (*p* < 0.0001), with the mean age of MSI cases being higher than that of MSS cases (75.43 versus 63.48 years). There was also a significant association with sex (*p* = 0.038), location (*p* = 0.015) and histological type (*p* = 0.033), with 24.5% of women, 27.1% of antrum tumors and 23.7% of intestinal-type tumors displaying MSI.

Correlation with the degree of differentiation, tumor size, lymph node involvement, metastases, stages and lymphatic, vascular or perineural involvement showed no significant results. However, of the 23 patients with MSI, 11 had no lymph node involvement, one had metastases and 15 were stage I/II.

Of the 43 patients who received chemotherapy, five were MSI (11.6%), while of the 99 patients who did not receive chemotherapy, 18 had MSI tumors (18.2%). Of those who died, 17.4% were MSI. Logistic regression of MSI, chemotherapy and survival showed no significant results, although of the five patients with MSI who had received chemotherapy, four had died (Table 4).

Two patients were both HER2-positive and MSI concurrently. Table 5 shows the clinical and pathological features of the gastric cancer patients showing both HER2-positive and MSI status, highlighting the match in most parameters.

## 4. Discussion

Gastric cancer is one of the leading causes of cancer and death globally [1,2,3,4,5,6,7,8,9,14,15,17], although its considerable heterogeneity hinders the individualization of patients that precision medicine would require [1,5,14,29,34,36]. For this reason, the traditional classifications have been superseded by the greater clinical utility of the new molecular classifications [28,30]. In this study, we examined a series of 142 GC cases, in which HER2 and MSI markers were determined and correlated with clinicopathological data to assess whether, as proposed, this could have an influence on treatment or prognosis [4,5,8,31,33].

Although recent advances in molecular biology advances have been made, they do not yet lead to a choice in treatment approach except in advanced disease with overexpression of HER2 [46].

The clinicopathological features of GC patients (Table 1) are: advanced mean age (65.41 years), predominance of men (62.7%), intestinal histological type (53.5%) and location in the corpus (40.8%) and antrum (41.5%), as well as stages II (33.1%) and III (32.4%). These data are comparable to those of previous observations [1,5,23,29,40], although in recent decades there has been a decrease in the intestinal type and distal location [47].

The proportion of HER2 positivity in our series was 13.4%, results that are very similar to those obtained by other authors in Europe and Asia [29,36,38], which would support the theory that no geographical differences exist [21,37]. Nonetheless, variable figures (6–38%) have been published according to the location and histology of the tumor [1,5,6,7,9,17,20,24]. Other factors, such as the type of sample (biopsy or surgical specimen) or the criteria for positivity, may also play a role [20,21,36,48]. In our study, we exclusively used gastrectomy specimens, and only cases with 3+ protein expression were considered positive.

The 16.2% MSI percentage is within the 15–30% range previously reported [1,4,6,23,28,30], although lower figures have also been described [29,40,43]. This variability could be due to patient and sample selection, intra-tumoral heterogeneity, as well as methodological and possibly geographic differences [3,40,43], with a higher prevalence in the Western population than in the Asian population [10].

### 4.1. HER2-Positive Status and Clinicopathological Features

The mean age of patients with HER2-positive GC in our study was 61 years, with more than twice as many men as women, as in other publications [36,37]. This type of tumor appeared mainly located in the corpus and antrum, in contrast to findings of other authors, which associate HER2 positivity with proximal location, especially in the gastroesophageal junction [1,21,37,38,48]. However, our data agree with those reported in one study [36], and a lack of correlation between HER2 status and location has also been communicated [43]. This discrepancy could be due to the low percentage of proximal neoplasms in our series, since tumors of the gastroesophageal junction and some tumors of the proximal stomach are classified as esophageal cancer [40,49].

There is a relevant association with histological type, with HER2-positive accounting for 21.1% of intestinal-type tumors, which coincides with published percentages of 13.7–34% [1,2,6,21,38]. Our data confirm that, as reported [36,37], more than 80% of patients with HER2-positive GC have intestinal-type tumors. The degree of differentiation, TNM classification, staging and lymphatic, vascular or perineural involvement have no or unclear correlation with HER2 overexpression [17,36,37,38]. Our study confirms these aspects, with the exception of metastases and staging, which do correlate, since five of the 13 patients in our series who had metastases were HER2-positive and the majority of cases were in advanced stages, possibly due to a greater aggressiveness of the tumor [19].

Regarding survival, although not statistically significant, it is important to note that 16.3% of the deceased had HER2-positive GC and that almost 75% of the HER2-positive patients died, which together with the data on metastasis and advanced stages would corroborate the poor prognosis associated with this type of GC, although a clear consensus is still lacking [1,5,6,19,36,38,48]. The correlation with the administration of chemotherapy could be related to the standard treatment with a trastuzumab-containing regimen [1,5,6,9], as HER2 status predicts prognosis and sensitivity to anti-HER2 agents [46,50].

### 4.2. MSI Status and Clinicopathological Features

The mean age of our patients with MSI was 75 years (10 years more for GC), similar to that of the TCGA study [28]; 21 of the 23 cases were older than 70 years, with a marked correlation that corroborates the connection between MSI and advanced age [3,23,29,31,40,43,51,52,53]. It has been noted that, despite the higher incidence of GC in men, the MSI type is more frequent in women [1,2,3,4,16,23,28,40,43,51,52,53]; this is demonstrated by its association with sex, as 13 of the 23 cases of MSI were women. The tumor appears to be associated with a distal or mid/lower location, mainly in the antrum, which is consistent with data from the literature [1,2,23,30,31,40,43,51,52,53].

Our results show that MSI GC is mainly of the intestinal type (78.3% of MSI cases), data similar to those of other authors [16,23,28,51], although different figures have also been published [2,29,30,40], which could be due to methodological and geographical differences [43,53]. The degree of differentiation, tumor size and lymphatic, vascular or perineural involvement were not associated with MSI status, as has been previously reported [23,40]. The same was found with lymph node involvement, metastases and stages. However, almost half of our MSI patients had no lymph node involvement, only one had metastases and two thirds had a low stage, which is in line with other studies [23,30,34,40,51,53] and could be considered clinically relevant and related to the prognosis.

No significant association was found between MSI status, perioperative chemotherapy and survival, although 80% of the patients with MSI who underwent chemotherapy died compared to 61% of those treated with surgery alone, and there was no such difference in patients with MSS. Of all GCs, MSI has been highlighted as having the best prognosis [10,22,28,30,33,40,51,52,53,54], especially in patients undergoing surgery alone, with a worse prognosis when neoadjuvant chemotherapy is used [1,4,7,40,41,42,43]. These data emphasize the prognostic and predictive role of the MSI marker, as has been noted [6,17,19] and, although further studies will be needed, it could be of interest to consider MSI determination before initiating chemotherapy, since, in these MSI cases (and also in EBV), chemotherapy does not improve survival [55]. Our study is retrospective, but it is possible that, in some cases of MSI GC, the use of chemotherapy could have been avoided.

Nevertheless, the low incidence of MSI GC indicates that the total number of cases studied continues to be low, so the debate remains open [56,57]. Additionally, the MSI and EBV subtypes of GC have been associated with a good response to immunotherapy [1,12,35,51,54,55,56,57]. This approach may be of therapeutic importance, due to the use of immune checkpoint inhibitors, although it has not yet been fully demonstrated in the first or second line [1,46]. Additionally, pembrolizumab monotherapy can only be considered in MSI patients who had previously received at least two lines of treatment, as shown by the high response rate achieved by these patients [58]. Although a high rate of PD-L1 expression has been reported in cases of MSI GC [1,6,9,19], this biomarker may be considered for the use of immune checkpoint inhibitors [59]. In any case, it is important to note that pembrolizumab in MSI cancer cases was the first treatment approved by the FDA according to the type of biomarker, regardless of the anatomical location of the tumor [30,34].

Two of the 142 patients showed HER2-positive and MSI status concurrently, with similar clinicopathological characteristics in both cases, although there were some differences. Both patients, male and female, deceased, were over 71 years of age and were not treated with adjuvant chemotherapy. The tumor was adenocarcinoma located in the antrum, of intestinal type, with G3 differentiation grade, and lymphatic, vascular and perineural involvement. One case was in stage IIA, showing no metastasis or lymph node involvement, and the other patient was in stage IV, exhibiting metastasis and lymph node implication. Several cases of patients showing HER2-positive and MSI/PD-L1-positive concomitant GC have been reported [60]. Moreover, a significant number of HER2-mutated and ERBB3-mutated samples associated with MSI have been found in metastatic colorectal cancer, theorizing the possibility of combining immunotherapy with anti-HER2 agents for these patients [61].

Molecular classification of the GC is a useful tool for treatment. It is necessary to identify the subgroups that can benefit the most from specific treatments and immunotherapy, together with strategies to avoid the immunosuppression that occurs in a high percentage of CGs [62]. Our study comprised limited casuistry and has only determined the HER2 and MSI subtypes, but the integration of molecular and clinicopathological data could help to develop targeted therapies and identify predictive and prognostic markers. Although, with the exception of HER2, the new biomarkers are not yet integrated into daily clinical practice, the importance of their determination should be emphasized, especially if we take into account that, as we have confirmed for HER2 and MSI, they are associated with specific clinicopathological features of the patients. HER2 testing should always be conducted to select patients with metastatic disease for specific treatment and MSI should be tested in advanced GC to predict the clinical benefit of immune checkpoint inhibitors [46]. The future of gastrointestinal oncology needs new research to establish different patient groups, early diagnosis and new therapeutic strategies. We are extending the characterization of HER2 and MSI molecular types to new GC patients and starting EBV determination through in situ hybridization. We hope that future studies on the integration of molecular and clinicopathological data in GC will contribute to the progress in the identification of prognostic and predictive biomarkers, as well as in the development of specific therapeutic strategies.

## 5. Conclusions

In conclusion, the general clinicopathological features of our GC patients are advanced age, male sex, intestinal type, proximal location and stages II-III. Tumors were HER2-positive in 13.4% and MSI in 16.2%, and they were associated with certain clinicopathological characteristics. The determination of HER2 and MSI status in GC is relevant for precision oncology. HER2 positivity was associated with intestinal type, metastasis and advanced tumor stages. More than twice as many HER2-positive GC patients were men, and most of them died. It was associated with a poor prognosis. Microsatellite instability was correlated with advanced age, female sex, distal location and intestinal type. There were few lymph node and distant metastases, with a predominance of early tumor stages. The percentage of deaths was lower if treated with surgery alone. It was associated with a better prognosis. The data supports the importance of determining HER2 and MSI status in GC, considering that they are not always routinely evaluated.

## Figures and Tables

**Table 1:** Clinical, pathological and molecular (HER2 positivity and MSI) features of the 142 patients with gastric cancer.

Features	Cases	Percentage%
Age (mean and range)	65.41	34–86
≤70	83	58.45%
≥71	59	41.55%
Molecular characteristics		
HER2-positive	19	13.40%
MSI	23	16.20%
Sex		
Male	89	62.70%
Female	53	37.30%
Location		
Cardia	23	16.20%
Fundus	1	0.70%
Corpus	58	40.80%
Antrum	59	41.50%
Histological type		
Intestinal	76	53.50%
Diffuse	57	40.10%
Mixed	9	6.40%
Pathological diagnosis		
Adenocarcinoma	104	73.20%
Signet ring cell carcinoma	34	23.90%
Small cell carcinoma	2	1.40%
Undifferentiated carcinoma	1	0.70%
Squamous cell carcinoma	1	0.70%
Degree of differentiation		
G1	18	13.00%
G2	46	33.30%
G3	74	53.60%
T		
Tis	6	4.20%
T1a/T1b	15	10.60%
T2	21	14.80%
T3	57	40.10%
T4a/T4b	43	30.30%
N		
N0	54	38.00%
N1	38	26.80%
N2	23	16.20%
N3a/N3b	27	19.00%
M		
M0	129	90.80%
M1	13	9.20%
Stage		
IA/IB	33	23.24%
IIA/IIB	47	33.10%
IIIA/IIIB/IIIC	46	32.40%
IV	16	11.26%
Lymphatic involvement		
Yes	64	45.10%
No	78	54.90%
Vascular involvement		
Yes	59	41.50%
No	83	58.50%
Perineural involvement		
Yes	48	33.80%
No	94	66.20%
Adjuvant chemotherapy		
Yes	43	30.30%
No	99	69.70%
Survival		
Living	56	39.40%
Deceased	86	60.60%

HER2: Human Epidermal Growth Receptor 2, MSI: Microsatellite instability.

**Table 2:** Clinical and pathological features of the 142 gastric cancer patients and correlation with HER2 status.

HER2-Positive (*n* = 19)			HER2-Negative (*n* = 123)		
Features	Cases	Percentage%	Cases	Percentage%	*p*
Age					0.085
Mean	61		66		
≤70	14	16.86%	70	84.33%	
≥71	5	8.47%	53	91.53%	
Sex					0.578
Male	13	14.60%	76	85.40%	
Female	6	11.30%	47	88.70%	
Location					0.041
Cardia	1	4.30%	22	95.70%	
Fundus	1	100%	0	0.0%	
Corpus	8	13.80%	50	86.20%	
Antrum	9	15.30%	50	84.70%	
Histological type					0.013
Intestinal	16	21.10%	60	78.90%	
Diffuse	2	3.50%	55	96.50%	
Mixed	1	11.10%	9	88.90%	
Pathological diagnosis					0.267
Adenocarcinoma	18	17.30%	86	82.70%	
Signet ring cell carcinoma	1	2.90%	33	97.10%	
Small cell carcinoma	0	0.00%	2	100%	
Undifferentiated carcinoma	0	0.00%	1	100%	
Squamous cell carcinoma	0	0.00%	1	100%	
Degree of differentiation					0.178
G1	0	0.00%	18	100%	
G2	8	17.40%	38	82.60%	
G3	11	14.90%	63	85.10%	
T					0.25
Tis	0	0.00%	6	100%	
T1a/T1b	0	0.00%	15	100%	
T2	1	4.80%	20	95.20%	
T3	11	19.30%	46	80.70%	
T4a/T4b	7	16.27%	36	83.73%	
N					0.455
N0	5	9.30%	49	90.70%	
N1	5	13.20%	33	86.80%	
N2	3	13.00%	20	87.00%	
N3a/N3b	6	22.20%	21	77.80%	
M					0.005
M0	14	10.90%	115	89.10%	
M1	5	38.50%	8	61.50%	
Stage					0.012
IA/IB	1	3.10%	32	96.90%	
IIA/IIB	6	12.80%	41	87.20%	
IIIA/IIIB/IIIC	6	13.00%	40	87.00%	
IV	6	37.50%	10	62.50%	
Lymphatic involvement				0.227	
Yes	11	17.20%	53	82.80%	
No	8	10.30%	70	89.70%	
Vascular involvement				0.292	
Yes	10	16.90%	49	83.10%	
No	9	10.80%	74	89.20%	
Perineural involvement				0.763	
Yes	7	14.60%	41	85.40%	
No	12	12.80%	82	87.20%	
Adjuvant chemotherapy				0.005	
Yes	11	25.60%	32	74.40%	
No	8	8.10%	91	91.90%	
Survival				0.209	
Living	5	8.90%	51	91.10%	
Deceased	14	16.30%	72	83.70%	

HER2: Human Epidermal Growth Receptor 2.

**Table 3:** Clinical and pathological features of the 142 gastric cancer patients and correlation with MSI status.

MSI (*n* = 23)			MSS (*n* = 119)		
Features	Cases	Percentage%	Cases	Percentage%	*p*
Age					<0.0001
Mean	75.43		63.48		
≤70	2	2.40%	81	97.60%	
≥71	21	35.59%	38	64.41%	
Sex					0.038
Male	10	11.20%	79	88.80%	
Female	13	24.50%	40	75.50%	
Location					0.015
Cardia	0	0.00%	23	100.00%	
Fundus	0	0.00%	1	100.00%	
Corpus	7	12.10%	51	89.70%	
Antrum	16	27.10%	43	72.90%	
Histological type					0.033
Intestinal	18	23.70%	58	76.30%	
Diffuse	4	7.00%	53	93.00%	
Mixed	1	11.10%	8	88.90%	
Pathological diagnosis					0.324
Adenocarcinoma	21	20.20%	83	79.80%	
Signet ring cell carcinoma	2	5.90%	32	94.10%	
Small cell carcinoma	0	0.00%	2	100.00%	
Undifferentiated carcinoma	0	0.00%	1	100.00%	
Squamous cell carcinoma	0	0.00%	1	100.00%	
Degree of differentiation					0.122
G1	6	33.30%	12	66.70%	
G2	6	13.00%	40	87.00%	
G3	11	14.90%	63	85.10%	
T					0.593
Tis	2	33.30%	4	66.70%	
T1a/T1b	2	13.30%	13	86.70%	
T2	5	23.80%	16	76.20%	
T3	7	12.30%	50	87.70%	
T4a/T4b	7	16.27%	36	83.73%	
N					0.647
N0	11	20.40%	43	79.60%	
N1	4	10.50%	34	89.50%	
N2	4	17.40%	19	82.60%	
N3a/N3b	4	14.80%	23	85.20%	
M					0.383
M0	22	17.10%	107	82.90%	
M1	1	7.70%	12	92.30%	
Stage					0.555
IA/IB	5	15.20%	28	84.80%	
IIA/IIB	10	21.30%	37	78.72%	
IIIA/IIIB/IIIC	7	15.20%	39	84.80%	
IV	1	6.30%	15	93.70%	
Lymphatic involvement				0.532	
Yes	9	14.10%	55	85.90%	
No	14	17.90%	64	82.10%	
Vascular involvement				0.797	
Yes	9	15.30%	50	84.70%	
No	14	16.90%	69	83.10%	
Perineural involvement				0.914	
Yes	8	16.70%	40	83.30%	
No	15	16.00%	79	84.00%	
Adjuvant chemotherapy				0.33	
Yes	5	11.60%	38	88.40%	
No	18	18.20%	81	81.80%	
Survival				0.618	
Living	8	14.30%	48	85.70%	
Deceased	15	17.40%	71	82.60%	

MSI: Microsatellite instability; MSS: Microsatellite stability.

**Table 4:** Association of MSI status with perioperative chemotherapy administration and survival. MSI: microsatellite instability; MSS: microsatellite stability.

MSI			MSS		
Status	Living	Deceased	Living	Deceased	*p*
Chemotherapy	1 (20%)	4 (80%)	15 (39.5%)	23 (60.5%)	0.397
Surgery alone	7 (38.9%)	11 (61.1%)	33 (40.7%)	48 (59.3%)	0.885

MSI: Microsatellite instability; MSS: Microsatellite stability.

**Table 5:** Clinical and pathological features of the gastric cancer patients showing both HER2-positive and MSI status.

Features	HER2-Positive and MSI (*n* = 2)	
	Case 1	Case 2
Age	76	74
Sex	Female	Male
Location	Antrum	Antrum
Histological type	Intestinal	Intestinal
Pathological diagnosis	Adenocarcinoma	Adenocarcinoma
Degree of differentiation	G3	G3
T	T3	T3
N	N3a	N0
M	M1	M0
Stage	IV	IIA
Lymphatic involvement	Yes	Yes
Vascular involvement	Yes	Yes
Perineural involvement	Yes	Yes
Adjuvant chemotherapy	No	No
Survival	Deceased	Deceased

HER2: Human Epidermal Growth Receptor 2, MSI: Microsatellite instability.

## Data Availability

The data that support the findings of this study are available from the corresponding author upon reasonable request.
